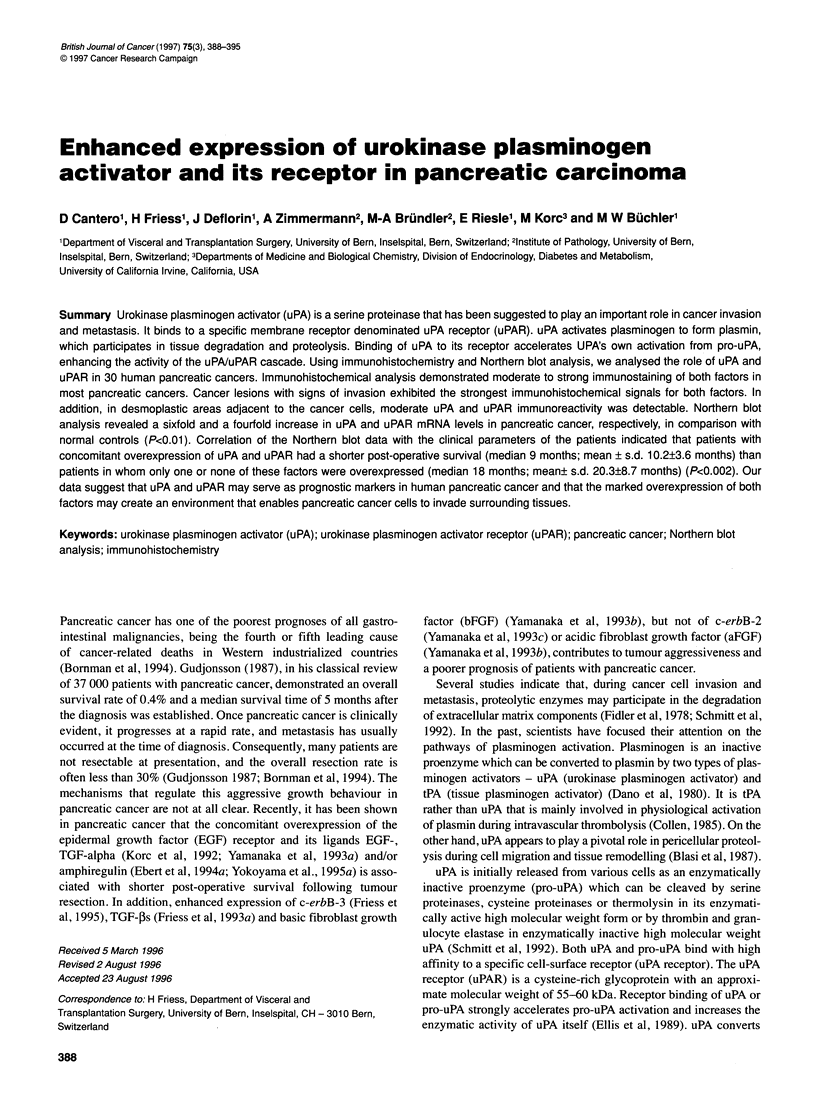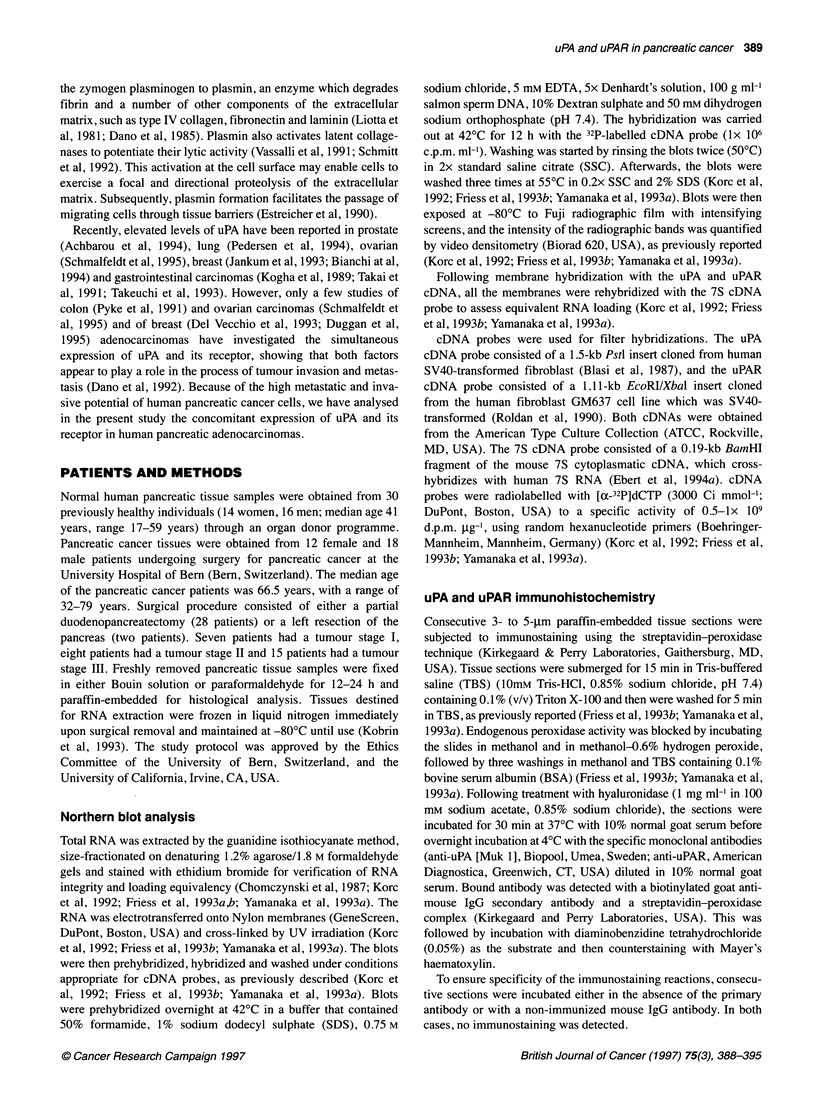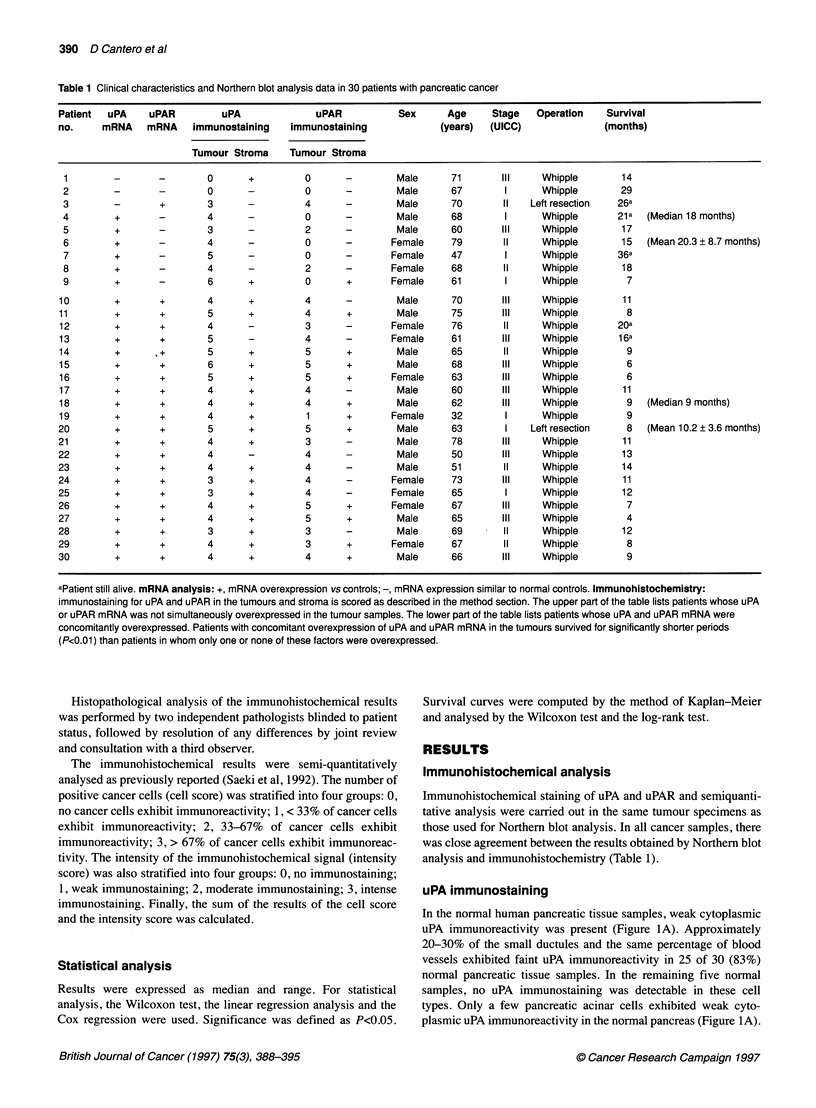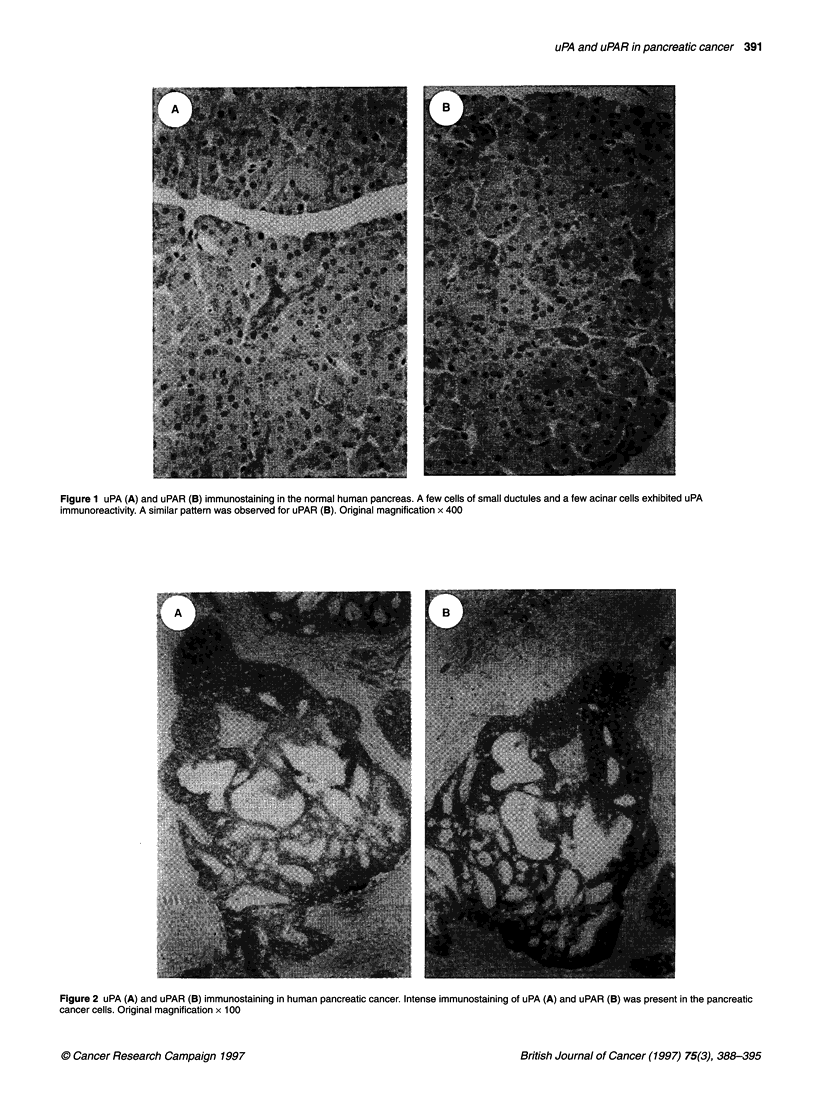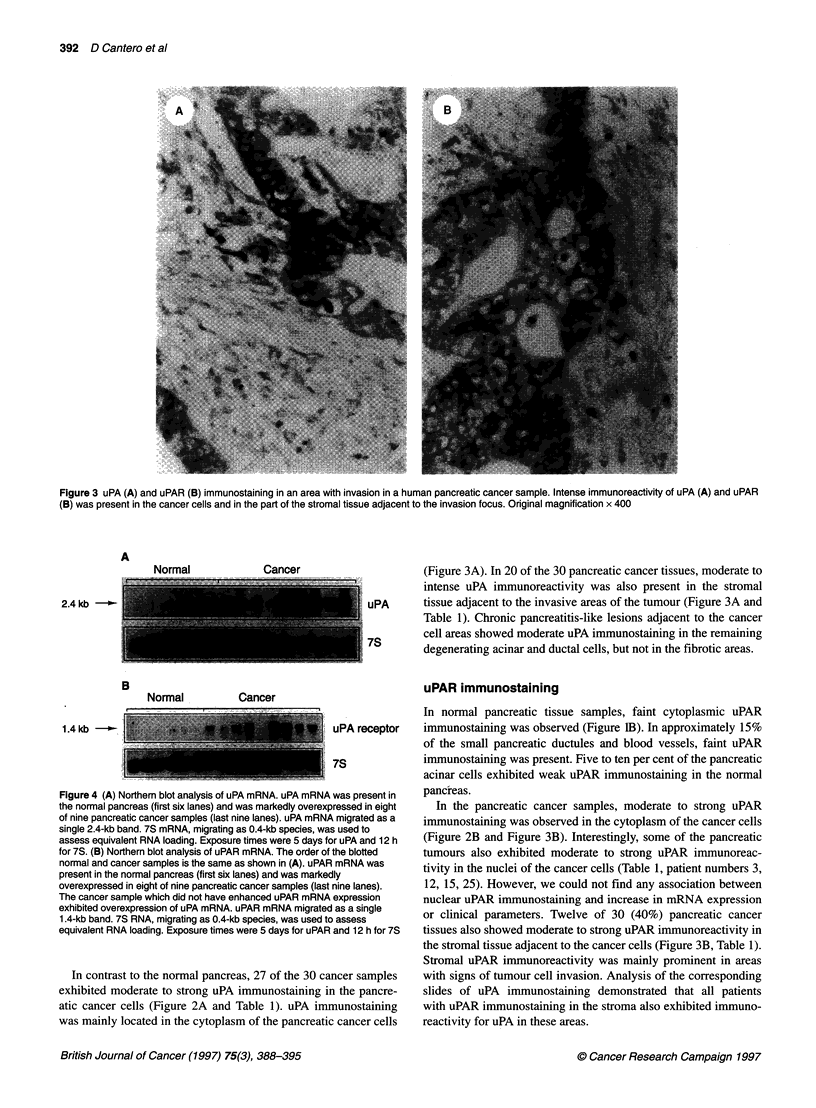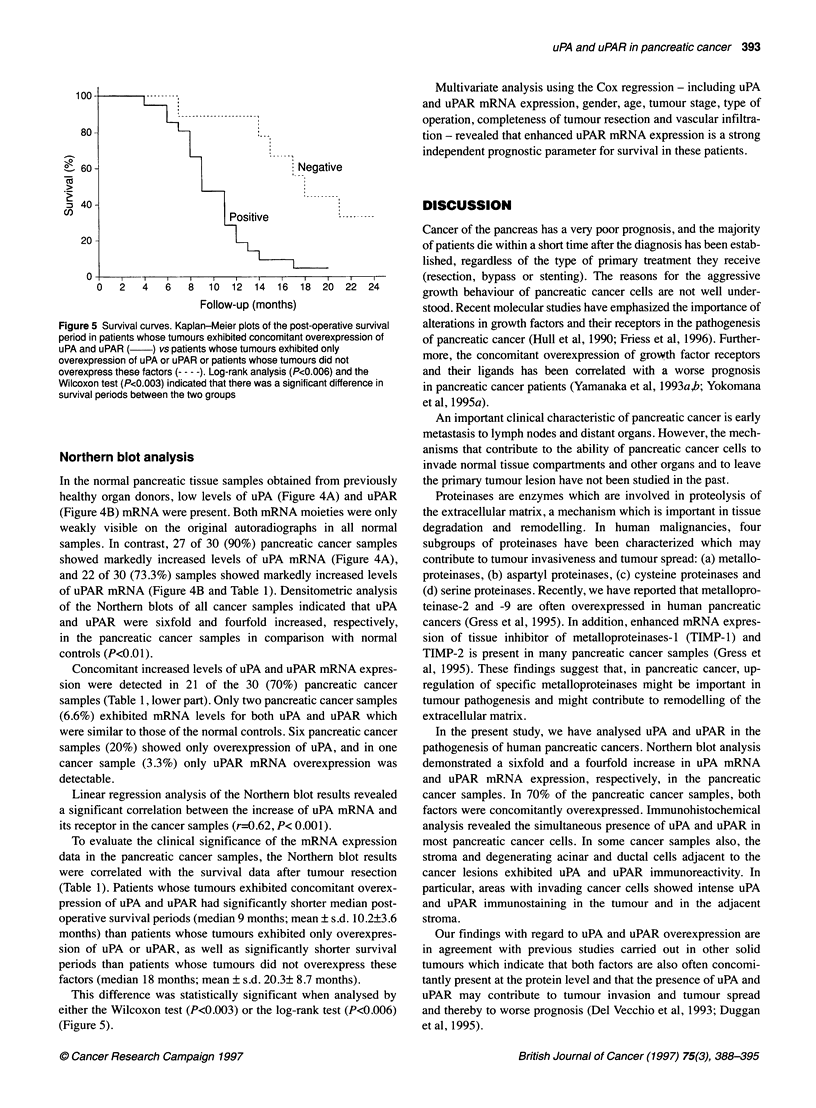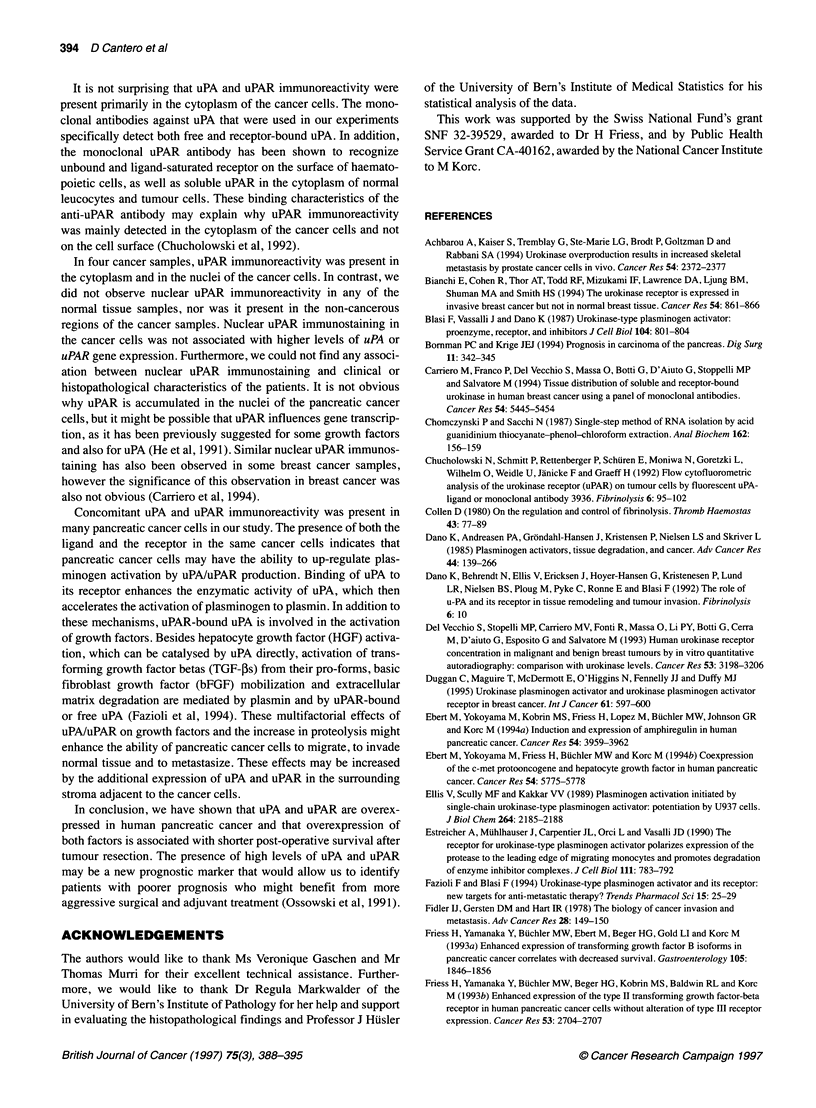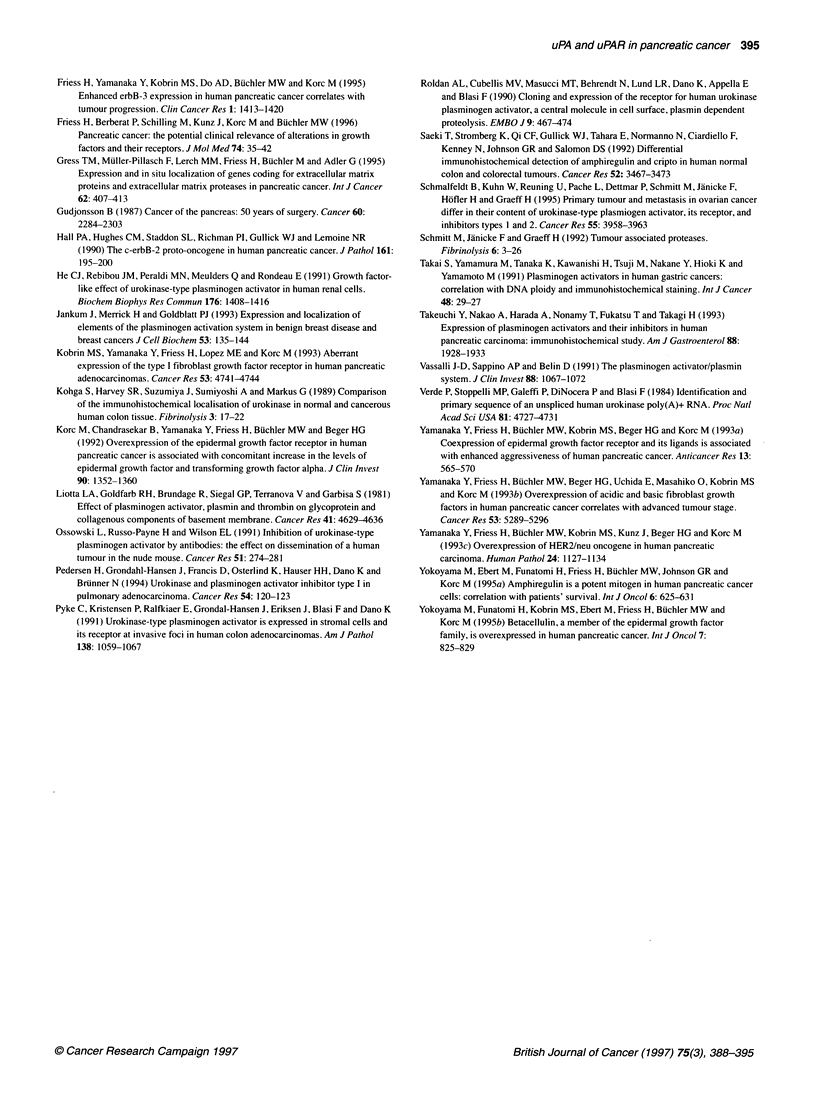# Enhanced expression of urokinase plasminogen activator and its receptor in pancreatic carcinoma.

**DOI:** 10.1038/bjc.1997.63

**Published:** 1997

**Authors:** D. Cantero, H. Friess, J. Deflorin, A. Zimmermann, M. A. Bründler, E. Riesle, M. Korc, M. W. Büchler

**Affiliations:** Department of Visceral and Transplantation Surgery, University of Bern, Inselspital, Switzerland.

## Abstract

**Images:**


British Journal of Cancer (1997) 75(3), 388–395
© 1997 Cancer Research Campaign

### Enhanced expression of urokinase plasminogen activator and its receptor in pancreatic carcinoma

**D Cantero[1], H Friess[1], J Deflorin[1], A Zimmermann[2], M-A Bründler[2], E Riesle[1], M Korc[3] and M W Büchler[1]**

[1]Department of Visceral and Transplantation Surgery, University of Bern, Inselspital, Bern, Switzerland; [2]Institute of Pathology, University of Bern, Inselspital, Bern, Switzerland; [3]Departments of Medicine and Biological Chemistry, Division of Endocrinology, Diabetes and Metabolism, University of California Irvine, California, USA

**Summary** Urokinase plasminogen activator (uPA) is a serine proteinase that has been suggested to play an important role in cancer invasion and metastasis. It binds to a specific membrane receptor denominated uPA receptor (uPAR). uPA activates plasminogen to form plasmin, which participates in tissue degradation and proteolysis. Binding of uPA to its receptor accelerates UPA's own activation from pro-uPA, enhancing the activity of the uPA/uPAR cascade. Using immunohistochemistry and Northern blot analysis, we analysed the role of uPA and uPAR in 30 human pancreatic cancers. Immunohistochemical analysis demonstrated moderate to strong immunostaining of both factors in most pancreatic cancers. Cancer lesions with signs of invasion exhibited the strongest immunohistochemical signals for both factors. In addition, in desmoplastic areas adjacent to the cancer cells, moderate uPA and uPAR immunoreactivity was detectable. Northern blot analysis revealed a sixfold and a fourfold increase in uPA and uPAR mRNA levels in pancreatic cancer, respectively, in comparison with normal controls ($P<0.01$). Correlation of the Northern blot data with the clinical parameters of the patients indicated that patients with concomitant overexpression of uPA and uPAR had a shorter post-operative survival (median 9 months; mean ± s.d. 10.2±3.6 months) than patients in whom only one or none of these factors were overexpressed (median 18 months; mean± s.d. 20.3±8.7 months) ($P<0.002$). Our data suggest that uPA and uPAR may serve as prognostic markers in human pancreatic cancer and that the marked overexpression of both factors may create an environment that enables pancreatic cancer cells to invade surrounding tissues.

**Keywords:** urokinase plasminogen activator (uPA); urokinase plasminogen activator receptor (uPAR); pancreatic cancer; Northern blot analysis; immunohistochemistry

Pancreatic cancer has one of the poorest prognoses of all gastrointestinal malignancies, being the fourth or fifth leading cause of cancer-related deaths in Western industrialized countries (Bornman et al, 1994). Gudjonsson (1987), in his classical review of 37 000 patients with pancreatic cancer, demonstrated an overall survival rate of 0.4% and a median survival time of 5 months after the diagnosis was established. Once pancreatic cancer is clinically evident, it progresses at a rapid rate, and metastasis has usually occurred at the time of diagnosis. Consequently, many patients are not resectable at presentation, and the overall resection rate is often less than 30% (Gudjonsson 1987; Bornman et al, 1994). The mechanisms that regulate this aggressive growth behaviour in pancreatic cancer are not at all clear. Recently, it has been shown in pancreatic cancer that the concomitant overexpression of the epidermal growth factor (EGF) receptor and its ligands EGF-, TGF-alpha (Korc et al, 1992; Yamanaka et al, 1993*a*) and/or amphiregulin (Ebert et al, 1994*a*; Yokoyama et al., 1995*a*) is associated with shorter post-operative survival following tumour resection. In addition, enhanced expression of c-*erb*B-3 (Friess et al, 1995), TGF-βs (Friess et al, 1993*a*) and basic fibroblast growth factor (bFGF) (Yamanaka et al, 1993*b*), but not of c-*erb*B-2 (Yamanaka et al, 1993*c*) or acidic fibroblast growth factor (aFGF) (Yamanaka et al, 1993*b*), contributes to tumour aggressiveness and a poorer prognosis of patients with pancreatic cancer.

*Received 5 March 1996*
*Revised 2 August 1996*
*Accepted 23 August 1996*

*Correspondence to:* H Friess, Department of Visceral and Transplantation Surgery, University of Bern, Inselspital, CH – 3010 Bern, Switzerland

Several studies indicate that, during cancer cell invasion and metastasis, proteolytic enzymes may participate in the degradation of extracellular matrix components (Fidler et al, 1978; Schmitt et al, 1992). In the past, scientists have focused their attention on the pathways of plasminogen activation. Plasminogen is an inactive proenzyme which can be converted to plasmin by two types of plasminogen activators – uPA (urokinase plasminogen activator) and tPA (tissue plasminogen activator) (Dano et al, 1980). It is tPA rather than uPA that is mainly involved in physiological activation of plasmin during intravascular thrombolysis (Collen, 1985). On the other hand, uPA appears to play a pivotal role in pericellular proteolysis during cell migration and tissue remodelling (Blasi et al, 1987).

uPA is initially released from various cells as an enzymatically inactive proenzyme (pro-uPA) which can be cleaved by serine proteinases, cysteine proteinases or thermolysin in its enzymatically active high molecular weight form or by thrombin and granulocyte elastase in enzymatically inactive high molecular weight uPA (Schmitt et al, 1992). Both uPA and pro-uPA bind with high affinity to a specific cell-surface receptor (uPA receptor). The uPA receptor (uPAR) is a cysteine-rich glycoprotein with an approximate molecular weight of 55–60 kDa. Receptor binding of uPA or pro-uPA strongly accelerates pro-uPA activation and increases the enzymatic activity of uPA itself (Ellis et al, 1989). uPA converts the zymogen plasminogen to plasmin, an enzyme which degrades fibrin and a number of other components of the extracellular matrix, such as type IV collagen, fibronectin and laminin (Liotta et al, 1981; Dano et al, 1985). Plasmin also activates latent collagenases to potentiate their lytic activity (Vassalli et al, 1991; Schmitt et al, 1992). This activation at the cell surface may enable cells to exercise a focal and directional proteolysis of the extracellular matrix. Subsequently, plasmin formation facilitates the passage of migrating cells through tissue barriers (Estreicher et al, 1990).

Recently, elevated levels of uPA have been reported in prostate (Achbarou et al, 1994), lung (Pedersen et al, 1994), ovarian (Schmalfeldt et al, 1995), breast (Jankum et al, 1993; Bianchi at al, 1994) and gastrointestinal carcinomas (Kogha et al, 1989; Takai et al, 1991; Takeuchi et al, 1993). However, only a few studies of colon (Pyke et al, 1991) and ovarian carcinomas (Schmalfeldt et al, 1995) and of breast (Del Vecchio et al, 1993; Duggan et al, 1995) adenocarcinomas have investigated the simultaneous expression of uPA and its receptor, showing that both factors appear to play a role in the process of tumour invasion and metastasis (Dano et al, 1992). Because of the high metastatic and invasive potential of human pancreatic cancer cells, we have analysed in the present study the concomitant expression of uPA and its receptor in human pancreatic adenocarcinomas.

### PATIENTS AND METHODS

Normal human pancreatic tissue samples were obtained from 30 previously healthy individuals (14 women, 16 men; median age 41 years, range 17–59 years) through an organ donor programme. Pancreatic cancer tissues were obtained from 12 female and 18 male patients undergoing surgery for pancreatic cancer at the University Hospital of Bern (Bern, Switzerland). The median age of the pancreatic cancer patients was 66.5 years, with a range of 32–79 years. Surgical procedure consisted of either a partial duodenopancreatectomy (28 patients) or a left resection of the pancreas (two patients). Seven patients had a tumour stage I, eight patients had a tumour stage II and 15 patients had a tumour stage III. Freshly removed pancreatic tissue samples were fixed in either Bouin solution or paraformaldehyde for 12–24 h and paraffin-embedded for histological analysis. Tissues destined for RNA extraction were frozen in liquid nitrogen immediately upon surgical removal and maintained at –80°C until use (Kobrin et al, 1993). The study protocol was approved by the Ethics Committee of the University of Bern, Switzerland, and the University of California, Irvine, CA, USA.

### Northern blot analysis

Total RNA was extracted by the guanidine isothiocyanate method, size-fractionated on denaturing 1.2% agarose/1.8 M formaldehyde gels and stained with ethidium bromide for verification of RNA integrity and loading equivalency (Chomczynski et al, 1987; Korc et al, 1992; Friess et al, 1993*a*,*b*; Yamanaka et al, 1993*a*). The RNA was electrotransferred onto Nylon membranes (GeneScreen, DuPont, Boston, USA) and cross-linked by UV irradiation (Korc et al, 1992; Friess et al, 1993*b*; Yamanaka et al, 1993*a*). The blots were then prehybridized, hybridized and washed under conditions appropriate for cDNA probes, as previously described (Korc et al, 1992; Friess et al, 1993*b*; Yamanaka et al, 1993*a*). Blots were prehybridized overnight at 42°C in a buffer that contained 50% formamide, 1% sodium dodecyl sulphate (SDS), 0.75 M sodium chloride, 5 mM EDTA, 5× Denhardt's solution, 100 g $ml^{-1}$ salmon sperm DNA, 10% Dextran sulphate and 50 mM dihydrogen sodium orthophosphate (pH 7.4). The hybridization was carried out at 42°C for 12 h with the $^{32}P$-labelled cDNA probe (1× $10^6$ c.p.m. $ml^{-1}$). Washing was started by rinsing the blots twice (50°C) in 2× standard saline citrate (SSC). Afterwards, the blots were washed three times at 55°C in 0.2× SSC and 2% SDS (Korc et al, 1992; Friess et al, 1993*b*; Yamanaka et al, 1993*a*). Blots were then exposed at –80°C to Fuji radiographic film with intensifying screens, and the intensity of the radiographic bands was quantified by video densitometry (Biorad 620, USA), as previously reported (Korc et al, 1992; Friess et al, 1993*b*; Yamanaka et al, 1993*a*).

Following membrane hybridization with the uPA and uPAR cDNA, all the membranes were rehybridized with the 7S cDNA probe to assess equivalent RNA loading (Korc et al, 1992; Friess et al, 1993*b*; Yamanaka et al, 1993*a*).

cDNA probes were used for filter hybridizations. The uPA cDNA probe consisted of a 1.5-kb *Pst*l insert cloned from human SV40-transformed fibroblast (Blasi et al, 1987), and the uPAR cDNA probe consisted of a 1.11-kb *Eco*RI/*Xba*l insert cloned from the human fibroblast GM637 cell line which was SV40-transformed (Roldan et al, 1990). Both cDNAs were obtained from the American Type Culture Collection (ATCC, Rockville, MD, USA). The 7S cDNA probe consisted of a 0.19-kb *Bam*HI fragment of the mouse 7S cytoplasmatic cDNA, which cross-hybridizes with human 7S RNA (Ebert et al, 1994*a*). cDNA probes were radiolabelled with [$\alpha$-$^{32}P$]dCTP (3000 Ci $mmol^{-1}$; DuPont, Boston, USA) to a specific activity of 0.5–1× $10^9$ d.p.m. $\mu g^{-1}$, using random hexanucleotide primers (Boehringer-Mannheim, Mannheim, Germany) (Korc et al, 1992; Friess et al, 1993*b*; Yamanaka et al, 1993*a*).

### uPA and uPAR immunohistochemistry

Consecutive 3- to 5-µm paraffin-embedded tissue sections were subjected to immunostaining using the streptavidin–peroxidase technique (Kirkegaard & Perry Laboratories, Gaithersburg, MD, USA). Tissue sections were submerged for 15 min in Tris-buffered saline (TBS) (10mM Tris-HCl, 0.85% sodium chloride, pH 7.4) containing 0.1% (v/v) Triton X-100 and then were washed for 5 min in TBS, as previously reported (Friess et al, 1993*b*; Yamanaka et al, 1993*a*). Endogenous peroxidase activity was blocked by incubating the slides in methanol and in methanol–0.6% hydrogen peroxide, followed by three washings in methanol and TBS containing 0.1% bovine serum albumin (BSA) (Friess et al, 1993*b*; Yamanaka et al, 1993*a*). Following treatment with hyaluronidase (1 mg $ml^{-1}$ in 100 mM sodium acetate, 0.85% sodium chloride), the sections were incubated for 30 min at 37°C with 10% normal goat serum before overnight incubation at 4°C with the specific monoclonal antibodies (anti-uPA [Muk 1], Biopool, Umea, Sweden; anti-uPAR, American Diagnostica, Greenwich, CT, USA) diluted in 10% normal goat serum. Bound antibody was detected with a biotinylated goat anti-mouse IgG secondary antibody and a streptavidin–peroxidase complex (Kirkegaard and Perry Laboratories, USA). This was followed by incubation with diaminobenzidine tetrahydrochloride (0.05%) as the substrate and then counterstaining with Mayer's haematoxylin.

To ensure specificity of the immunostaining reactions, consecutive sections were incubated either in the absence of the primary antibody or with a non-immunized mouse IgG antibody. In both cases, no immunostaining was detected.

© Cancer Research Campaign 1997

**Table 1** Clinical characteristics and Northern blot analysis data in 30 patients with pancreatic cancer

| Patient no. | uPA mRNA | uPAR mRNA | uPA immunostaining | | uPAR immunostaining | | Sex | Age (years) | Stage (UICC) | Operation | Survival (months) | |
|---|---|---|---|---|---|---|---|---|---|---|---|---|
| | | | Tumour | Stroma | Tumour | Stroma | | | | | | |
| 1 | – | – | 0 | + | 0 | – | Male | 71 | III | Whipple | 14 | |
| 2 | – | – | 0 | – | 0 | – | Male | 67 | I | Whipple | 29 | |
| 3 | – | + | 3 | – | 4 | – | Male | 70 | II | Left resection | 26[a] | |
| 4 | + | – | 4 | – | 0 | – | Male | 68 | I | Whipple | 21[a] | (Median 18 months) |
| 5 | + | – | 3 | – | 2 | – | Male | 60 | III | Whipple | 17 | |
| 6 | + | – | 4 | – | 0 | – | Female | 79 | II | Whipple | 15 | (Mean 20.3 ± 8.7 months) |
| 7 | + | – | 5 | – | 0 | – | Female | 47 | I | Whipple | 36[a] | |
| 8 | + | – | 4 | – | 2 | – | Female | 68 | II | Whipple | 18 | |
| 9 | + | – | 6 | + | 0 | + | Female | 61 | I | Whipple | 7 | |
| 10 | + | + | 4 | + | 4 | – | Male | 70 | III | Whipple | 11 | |
| 11 | + | + | 5 | + | 4 | + | Male | 75 | III | Whipple | 8 | |
| 12 | + | + | 4 | – | 3 | – | Female | 76 | II | Whipple | 20[a] | |
| 13 | + | + | 5 | – | 4 | – | Female | 61 | III | Whipple | 16[a] | |
| 14 | + | + | 5 | + | 5 | + | Male | 65 | II | Whipple | 9 | |
| 15 | + | + | 6 | + | 5 | + | Male | 68 | III | Whipple | 6 | |
| 16 | + | + | 5 | + | 5 | + | Female | 63 | III | Whipple | 6 | |
| 17 | + | + | 4 | + | 4 | – | Male | 60 | III | Whipple | 11 | |
| 18 | + | + | 4 | + | 4 | + | Male | 62 | III | Whipple | 9 | (Median 9 months) |
| 19 | + | + | 4 | + | 1 | + | Female | 32 | I | Whipple | 9 | |
| 20 | + | + | 5 | + | 5 | + | Male | 63 | I | Left resection | 8 | (Mean 10.2 ± 3.6 months) |
| 21 | + | + | 4 | + | 3 | – | Male | 78 | III | Whipple | 11 | |
| 22 | + | + | 4 | – | 4 | – | Male | 50 | III | Whipple | 13 | |
| 23 | + | + | 4 | + | 4 | – | Male | 51 | II | Whipple | 14 | |
| 24 | + | + | 3 | + | 4 | – | Female | 73 | III | Whipple | 11 | |
| 25 | + | + | 3 | + | 4 | – | Female | 65 | I | Whipple | 12 | |
| 26 | + | + | 4 | + | 5 | + | Female | 67 | III | Whipple | 7 | |
| 27 | + | + | 4 | + | 5 | + | Male | 65 | III | Whipple | 4 | |
| 28 | + | + | 3 | + | 3 | – | Male | 69 | II | Whipple | 12 | |
| 29 | + | + | 4 | + | 3 | + | Female | 67 | II | Whipple | 8 | |
| 30 | + | + | 4 | + | 4 | + | Male | 66 | III | Whipple | 9 | |

[a]Patient still alive. **mRNA analysis:** +, mRNA overexpression *vs* controls; –, mRNA expression similar to normal controls. **Immunohistochemistry:** immunostaining for uPA and uPAR in the tumours and stroma is scored as described in the method section. The upper part of the table lists patients whose uPA or uPAR mRNA was not simultaneously overexpressed in the tumour samples. The lower part of the table lists patients whose uPA and uPAR mRNA were concomitantly overexpressed. Patients with concomitant overexpression of uPA and uPAR mRNA in the tumours survived for significantly shorter periods ($P$<0.01) than patients in whom only one or none of these factors were overexpressed.

Histopathological analysis of the immunohistochemical results was performed by two independent pathologists blinded to patient status, followed by resolution of any differences by joint review and consultation with a third observer.

The immunohistochemical results were semi-quantitatively analysed as previously reported (Saeki et al, 1992). The number of positive cancer cells (cell score) was stratified into four groups: 0, no cancer cells exhibit immunoreactivity; 1, < 33% of cancer cells exhibit immunoreactivity; 2, 33–67% of cancer cells exhibit immunoreactivity; 3, > 67% of cancer cells exhibit immunoreactivity. The intensity of the immunohistochemical signal (intensity score) was also stratified into four groups: 0, no immunostaining; 1, weak immunostaining; 2, moderate immunostaining; 3, intense immunostaining. Finally, the sum of the results of the cell score and the intensity score was calculated.

### Statistical analysis

Results were expressed as median and range. For statistical analysis, the Wilcoxon test, the linear regression analysis and the Cox regression were used. Significance was defined as $P$<0.05. Survival curves were computed by the method of Kaplan–Meier and analysed by the Wilcoxon test and the log-rank test.

### RESULTS

### Immunohistochemical analysis

Immunohistochemical staining of uPA and uPAR and semiquantitative analysis were carried out in the same tumour specimens as those used for Northern blot analysis. In all cancer samples, there was close agreement between the results obtained by Northern blot analysis and immunohistochemistry (Table 1).

### uPA immunostaining

In the normal human pancreatic tissue samples, weak cytoplasmic uPA immunoreactivity was present (Figure 1A). Approximately 20–30% of the small ductules and the same percentage of blood vessels exhibited faint uPA immunoreactivity in 25 of 30 (83%) normal pancreatic tissue samples. In the remaining five normal samples, no uPA immunostaining was detectable in these cell types. Only a few pancreatic acinar cells exhibited weak cytoplasmic uPA immunoreactivity in the normal pancreas (Figure 1A).

© Cancer Research Campaign 1997

**Figure 1** uPA (**A**) and uPAR (**B**) immunostaining in the normal human pancreas. A few cells of small ductules and a few acinar cells exhibited uPA immunoreactivity. A similar pattern was observed for uPAR (**B**). Original magnification × 400

**Figure 2** uPA (**A**) and uPAR (**B**) immunostaining in human pancreatic cancer. Intense immunostaining of uPA (**A**) and uPAR (**B**) was present in the pancreatic cancer cells. Original magnification × 100

© Cancer Research Campaign 1997

Figure 3 uPA (A) and uPAR (B) immunostaining in an area with invasion in a human pancreatic cancer sample. Intense immunoreactivity of uPA (A) and uPAR (B) was present in the cancer cells and in the part of the stromal tissue adjacent to the invasion focus. Original magnification × 400

Figure 4 (A) Northern blot analysis of uPA mRNA. uPA mRNA was present in the normal pancreas (first six lanes) and was markedly overexpressed in eight of nine pancreatic cancer samples (last nine lanes). uPA mRNA migrated as a single 2.4-kb band. 7S mRNA, migrating as 0.4-kb species, was used to assess equivalent RNA loading. Exposure times were 5 days for uPA and 12 h for 7S. (B) Northern blot analysis of uPAR mRNA. The order of the blotted normal and cancer samples is the same as shown in (A). uPAR mRNA was present in the normal pancreas (first six lanes) and was markedly overexpressed in eight of nine pancreatic cancer samples (last nine lanes). The cancer sample which did not have enhanced uPAR mRNA expression exhibited overexpression of uPA mRNA. uPAR mRNA migrated as a single 1.4-kb band. 7S RNA, migrating as 0.4-kb species, was used to assess equivalent RNA loading. Exposure times were 5 days for uPAR and 12 h for 7S

In contrast to the normal pancreas, 27 of the 30 cancer samples exhibited moderate to strong uPA immunostaining in the pancreatic cancer cells (Figure 2A and Table 1). uPA immunostaining was mainly located in the cytoplasm of the pancreatic cancer cells (Figure 3A). In 20 of the 30 pancreatic cancer tissues, moderate to intense uPA immunoreactivity was also present in the stromal tissue adjacent to the invasive areas of the tumour (Figure 3A and Table 1). Chronic pancreatitis-like lesions adjacent to the cancer cell areas showed moderate uPA immunostaining in the remaining degenerating acinar and ductal cells, but not in the fibrotic areas.

### uPAR immunostaining

In normal pancreatic tissue samples, faint cytoplasmic uPAR immunostaining was observed (Figure lB). In approximately 15% of the small pancreatic ductules and blood vessels, faint uPAR immunostaining was present. Five to ten per cent of the pancreatic acinar cells exhibited weak uPAR immunostaining in the normal pancreas.

In the pancreatic cancer samples, moderate to strong uPAR immunostaining was observed in the cytoplasm of the cancer cells (Figure 2B and Figure 3B). Interestingly, some of the pancreatic tumours also exhibited moderate to strong uPAR immunoreactivity in the nuclei of the cancer cells (Table 1, patient numbers 3, 12, 15, 25). However, we could not find any association between nuclear uPAR immunostaining and increase in mRNA expression or clinical parameters. Twelve of 30 (40%) pancreatic cancer tissues also showed moderate to strong uPAR immunoreactivity in the stromal tissue adjacent to the cancer cells (Figure 3B, Table 1). Stromal uPAR immunoreactivity was mainly prominent in areas with signs of tumour cell invasion. Analysis of the corresponding slides of uPA immunostaining demonstrated that all patients with uPAR immunostaining in the stroma also exhibited immunoreactivity for uPA in these areas.

© Cancer Research Campaign 1997

**Figure 5** Survival curves. Kaplan–Meier plots of the post-operative survival period in patients whose tumours exhibited concomitant overexpression of uPA and uPAR (——) *vs* patients whose tumours exhibited only overexpression of uPA or uPAR or patients whose tumours did not overexpress these factors (- - - -). Log-rank analysis ($P$<0.006) and the Wilcoxon test ($P$<0.003) indicated that there was a significant difference in survival periods between the two groups

### Northern blot analysis

In the normal pancreatic tissue samples obtained from previously healthy organ donors, low levels of uPA (Figure 4A) and uPAR (Figure 4B) mRNA were present. Both mRNA moieties were only weakly visible on the original autoradiographs in all normal samples. In contrast, 27 of 30 (90%) pancreatic cancer samples showed markedly increased levels of uPA mRNA (Figure 4A), and 22 of 30 (73.3%) samples showed markedly increased levels of uPAR mRNA (Figure 4B and Table 1). Densitometric analysis of the Northern blots of all cancer samples indicated that uPA and uPAR were sixfold and fourfold increased, respectively, in the pancreatic cancer samples in comparison with normal controls ($P$<0.01).

Concomitant increased levels of uPA and uPAR mRNA expression were detected in 21 of the 30 (70%) pancreatic cancer samples (Table 1, lower part). Only two pancreatic cancer samples (6.6%) exhibited mRNA levels for both uPA and uPAR which were similar to those of the normal controls. Six pancreatic cancer samples (20%) showed only overexpression of uPA, and in one cancer sample (3.3%) only uPAR mRNA overexpression was detectable.

Linear regression analysis of the Northern blot results revealed a significant correlation between the increase of uPA mRNA and its receptor in the cancer samples ($r$=0.62, $P$< 0.001).

To evaluate the clinical significance of the mRNA expression data in the pancreatic cancer samples, the Northern blot results were correlated with the survival data after tumour resection (Table 1). Patients whose tumours exhibited concomitant overexpression of uPA and uPAR had significantly shorter median post-operative survival periods (median 9 months; mean ± s.d. 10.2±3.6 months) than patients whose tumours exhibited only overexpression of uPA or uPAR, as well as significantly shorter survival periods than patients whose tumours did not overexpress these factors (median 18 months; mean ± s.d. 20.3± 8.7 months).

This difference was statistically significant when analysed by either the Wilcoxon test ($P$<0.003) or the log-rank test ($P$<0.006) (Figure 5).

Multivariate analysis using the Cox regression – including uPA and uPAR mRNA expression, gender, age, tumour stage, type of operation, completeness of tumour resection and vascular infiltration – revealed that enhanced uPAR mRNA expression is a strong independent prognostic parameter for survival in these patients.

### DISCUSSION

Cancer of the pancreas has a very poor prognosis, and the majority of patients die within a short time after the diagnosis has been established, regardless of the type of primary treatment they receive (resection, bypass or stenting). The reasons for the aggressive growth behaviour of pancreatic cancer cells are not well understood. Recent molecular studies have emphasized the importance of alterations in growth factors and their receptors in the pathogenesis of pancreatic cancer (Hull et al, 1990; Friess et al, 1996). Furthermore, the concomitant overexpression of growth factor receptors and their ligands has been correlated with a worse prognosis in pancreatic cancer patients (Yamanaka et al, 1993*a*,*b*; Yokomana et al, 1995*a*).

An important clinical characteristic of pancreatic cancer is early metastasis to lymph nodes and distant organs. However, the mechanisms that contribute to the ability of pancreatic cancer cells to invade normal tissue compartments and other organs and to leave the primary tumour lesion have not been studied in the past.

Proteinases are enzymes which are involved in proteolysis of the extracellular matrix, a mechanism which is important in tissue degradation and remodelling. In human malignancies, four subgroups of proteinases have been characterized which may contribute to tumour invasiveness and tumour spread: (a) metalloproteinases, (b) aspartyl proteinases, (c) cysteine proteinases and (d) serine proteinases. Recently, we have reported that metalloproteinase-2 and -9 are often overexpressed in human pancreatic cancers (Gress et al, 1995). In addition, enhanced mRNA expression of tissue inhibitor of metalloproteinases-1 (TIMP-1) and TIMP-2 is present in many pancreatic cancer samples (Gress et al, 1995). These findings suggest that, in pancreatic cancer, upregulation of specific metalloproteinases might be important in tumour pathogenesis and might contribute to remodelling of the extracellular matrix.

In the present study, we have analysed uPA and uPAR in the pathogenesis of human pancreatic cancers. Northern blot analysis demonstrated a sixfold and a fourfold increase in uPA mRNA and uPAR mRNA expression, respectively, in the pancreatic cancer samples. In 70% of the pancreatic cancer samples, both factors were concomitantly overexpressed. Immunohistochemical analysis revealed the simultaneous presence of uPA and uPAR in most pancreatic cancer cells. In some cancer samples also, the stroma and degenerating acinar and ductal cells adjacent to the cancer lesions exhibited uPA and uPAR immunoreactivity. In particular, areas with invading cancer cells showed intense uPA and uPAR immunostaining in the tumour and in the adjacent stroma.

Our findings with regard to uPA and uPAR overexpression are in agreement with previous studies carried out in other solid tumours which indicate that both factors are also often concomitantly present at the protein level and that the presence of uPA and uPAR may contribute to tumour invasion and tumour spread and thereby to worse prognosis (Del Vecchio et al, 1993; Duggan et al, 1995).

© Cancer Research Campaign 1997

It is not surprising that uPA and uPAR immunoreactivity were present primarily in the cytoplasm of the cancer cells. The monoclonal antibodies against uPA that were used in our experiments specifically detect both free and receptor-bound uPA. In addition, the monoclonal uPAR antibody has been shown to recognize unbound and ligand-saturated receptor on the surface of haematopoietic cells, as well as soluble uPAR in the cytoplasm of normal leucocytes and tumour cells. These binding characteristics of the anti-uPAR antibody may explain why uPAR immunoreactivity was mainly detected in the cytoplasm of the cancer cells and not on the cell surface (Chucholowski et al, 1992).

In four cancer samples, uPAR immunoreactivity was present in the cytoplasm and in the nuclei of the cancer cells. In contrast, we did not observe nuclear uPAR immunoreactivity in any of the normal tissue samples, nor was it present in the non-cancerous regions of the cancer samples. Nuclear uPAR immunostaining in the cancer cells was not associated with higher levels of *uPA* or *uPAR* gene expression. Furthermore, we could not find any association between nuclear uPAR immunostaining and clinical or histopathological characteristics of the patients. It is not obvious why uPAR is accumulated in the nuclei of the pancreatic cancer cells, but it might be possible that uPAR influences gene transcription, as it has been previously suggested for some growth factors and also for uPA (He et al, 1991). Similar nuclear uPAR immunostaining has also been observed in some breast cancer samples, however the significance of this observation in breast cancer was also not obvious (Carriero et al, 1994).

Concomitant uPA and uPAR immunoreactivity was present in many pancreatic cancer cells in our study. The presence of both the ligand and the receptor in the same cancer cells indicates that pancreatic cancer cells may have the ability to up-regulate plasminogen activation by uPA/uPAR production. Binding of uPA to its receptor enhances the enzymatic activity of uPA, which then accelerates the activation of plasminogen to plasmin. In addition to these mechanisms, uPAR-bound uPA is involved in the activation of growth factors. Besides hepatocyte growth factor (HGF) activation, which can be catalysed by uPA directly, activation of transforming growth factor betas (TGF-βs) from their pro-forms, basic fibroblast growth factor (bFGF) mobilization and extracellular matrix degradation are mediated by plasmin and by uPAR-bound or free uPA (Fazioli et al, 1994). These multifactorial effects of uPA/uPAR on growth factors and the increase in proteolysis might enhance the ability of pancreatic cancer cells to migrate, to invade normal tissue and to metastasize. These effects may be increased by the additional expression of uPA and uPAR in the surrounding stroma adjacent to the cancer cells.

In conclusion, we have shown that uPA and uPAR are overexpressed in human pancreatic cancer and that overexpression of both factors is associated with shorter post-operative survival after tumour resection. The presence of high levels of uPA and uPAR may be a new prognostic marker that would allow us to identify patients with poorer prognosis who might benefit from more aggressive surgical and adjuvant treatment (Ossowski et al, 1991).

### ACKNOWLEDGEMENTS

The authors would like to thank Ms Veronique Gaschen and Mr Thomas Murri for their excellent technical assistance. Furthermore, we would like to thank Dr Regula Markwalder of the University of Bern's Institute of Pathology for her help and support in evaluating the histopathological findings and Professor J Hüsler of the University of Bern's Institute of Medical Statistics for his statistical analysis of the data.

This work was supported by the Swiss National Fund's grant SNF 32-39529, awarded to Dr H Friess, and by Public Health Service Grant CA-40162, awarded by the National Cancer Institute to M Korc.

### REFERENCES

Achbarou A, Kaiser S, Tremblay G, Ste-Marie LG, Brodt P, Goltzman D and Rabbani SA (1994) Urokinase overproduction results in increased skeletal metastasis by prostate cancer cells in vivo. *Cancer Res* **54**: 2372–2377

Bianchi E, Cohen R, Thor AT, Todd RF, Mizukami IF, Lawrence DA, Ljung BM, Shuman MA and Smith HS (1994) The urokinase receptor is expressed in invasive breast cancer but not in normal breast tissue. *Cancer Res* **54**: 861–866

Blasi F, Vassalli J and Dano K (1987) Urokinase-type plasminogen activator: proenzyme, receptor, and inhibitors *J Cell Biol* **104**: 801–804

Bornman PC and Krige JEJ (1994) Prognosis in carcinoma of the pancreas. *Dig Surg* **11**: 342–345

Carriero M, Franco P, Del Vecchio S, Massa O, Botti G, D'Aiuto G, Stoppelli MP and Salvatore M (1994) Tissue distribution of soluble and receptor-bound urokinase in human breast cancer using a panel of monoclonal antibodies. *Cancer Res* **54**: 5445–5454

Chomczynski P and Sacchi N (1987) Single-step method of RNA isolation by acid guanidinium thiocyanate–phenol–chloroform extraction. *Anal Biochem* **162**: 156–159

Chucholowski N, Schmitt P, Rettenberger P, Schüren E, Moniwa N, Goretzki L, Wilhelm O, Weidle U, Jänicke F and Graeff H (1992) Flow cytofluorometric analysis of the urokinase receptor (uPAR) on tumour cells by fluorescent uPA-ligand or monoclonal antibody 3936. *Fibrinolysis* **6**: 95–102

Collen D (1980) On the regulation and control of fibrinolysis. *Thromb Haemostas* **43**: 77–89

Dano K, Andreasen PA, Gröndahl-Hansen J, Kristensen P, Nielsen LS and Skriver L (1985) Plasminogen activators, tissue degradation, and cancer. *Adv Cancer Res* **44**: 139–266

Dano K, Behrendt N, Ellis V, Ericksen J, Hoyer-Hansen G, Kristenesen P, Lund LR, Nielsen BS, Ploug M, Pyke C, Ronne E and Blasi F (1992) The role of u-PA and its receptor in tissue remodeling and tumour invasion. *Fibrinolysis* **6**: 10

Del Vecchio S, Stopelli MP, Carriero MV, Fonti R, Massa O, Li PY, Botti G, Cerra M, D'aiuto G, Esposito G and Salvatore M (1993) Human urokinase receptor concentration in malignant and benign breast tumours by in vitro quantitative autoradiography: comparison with urokinase levels. *Cancer Res* **53**: 3198–3206

Duggan C, Maguire T, McDermott E, O'Higgins N, Fennelly JJ and Duffy MJ (1995) Urokinase plasminogen activator and urokinase plasminogen activator receptor in breast cancer. *Int J Cancer* **61**: 597–600

Ebert M, Yokoyama M, Kobrin MS, Friess H, Lopez M, Büchler MW, Johnson GR and Korc M (1994*a*) Induction and expression of amphiregulin in human pancreatic cancer. *Cancer Res* **54**: 3959–3962

Ebert M, Yokoyama M, Friess H, Büchler MW and Korc M (1994*b*) Coexpression of the c-met protooncogene and hepatocyte growth factor in human pancreatic cancer. *Cancer Res* **54**: 5775–5778

Ellis V, Scully MF and Kakkar VV (1989) Plasminogen activation initiated by single-chain urokinase-type plasminogen activator: potentiation by U937 cells. *J Biol Chem* **264**: 2185–2188

Estreicher A, Mühlhauser J, Carpentier JL, Orci L and Vasalli JD (1990) The receptor for urokinase-type plasminogen activator polarizes expression of the protease to the leading edge of migrating monocytes and promotes degradation of enzyme inhibitor complexes. *J Cell Biol* **111**: 783–792

Fazioli F and Blasi F (1994) Urokinase-type plasminogen activator and its receptor: new targets for anti-metastatic therapy? *Trends Pharmacol Sci* **15**: 25–29

Fidler IJ, Gersten DM and Hart IR (1978) The biology of cancer invasion and metastasis. *Adv Cancer Res* **28**: 149–150

Friess H, Yamanaka Y, Büchler MW, Ebert M, Beger HG, Gold LI and Korc M (1993*a*) Enhanced expression of transforming growth factor B isoforms in pancreatic cancer correlates with decreased survival. *Gastroenterology* **105**: 1846–1856

Friess H, Yamanaka Y, Büchler MW, Beger HG, Kobrin MS, Baldwin RL and Korc M (1993*b*) Enhanced expression of the type II transforming growth factor-beta receptor in human pancreatic cancer cells without alteration of type III receptor expression. *Cancer Res* **53**: 2704–2707

© Cancer Research Campaign 1997

Friess H, Yamanaka Y, Kobrin MS, Do AD, Büchler MW and Korc M (1995) Enhanced erbB-3 expression in human pancreatic cancer correlates with tumour progression. *Clin Cancer Res* **1**: 1413–1420

Friess H, Berberat P, Schilling M, Kunz J, Korc M and Büchler MW (1996) Pancreatic cancer: the potential clinical relevance of alterations in growth factors and their receptors. *J Mol Med* **74**: 35–42

Gress TM, Müller-Pillasch F, Lerch MM, Friess H, Büchler M and Adler G (1995) Expression and in situ localization of genes coding for extracellular matrix proteins and extracellular matrix proteases in pancreatic cancer. *Int J Cancer* **62**: 407–413

Gudjonsson B (1987) Cancer of the pancreas: 50 years of surgery. *Cancer* **60**: 2284–2303

Hall PA, Hughes CM, Staddon SL, Richman PI, Gullick WJ and Lemoine NR (1990) The c-erbB-2 proto-oncogene in human pancreatic cancer. *J Pathol* **161**: 195–200

He CJ, Rebibou JM, Peraldi MN, Meulders Q and Rondeau E (1991) Growth factor-like effect of urokinase-type plasminogen activator in human renal cells. *Biochem Biophys Res Commun* **176**: 1408–1416

Jankum J, Merrick H and Goldblatt PJ (1993) Expression and localization of elements of the plasminogen activation system in benign breast disease and breast cancers *J Cell Biochem* **53**: 135–144

Kobrin MS, Yamanaka Y, Friess H, Lopez ME and Korc M (1993) Aberrant expression of the type I fibroblast growth factor receptor in human pancreatic adenocarcinomas. *Cancer Res* **53**: 4741–4744

Kohga S, Harvey SR, Suzumiya J, Sumiyoshi A and Markus G (1989) Comparison of the immunohistochemical localisation of urokinase in normal and cancerous human colon tissue. *Fibrinolysis* **3**: 17–22

Korc M, Chandrasekar B, Yamanaka Y, Friess H, Büchler MW and Beger HG (1992) Overexpression of the epidermal growth factor receptor in human pancreatic cancer is associated with concomitant increase in the levels of epidermal growth factor and transforming growth factor alpha. *J Clin Invest* **90**: 1352–1360

Liotta LA, Goldfarb RH, Brundage R, Siegal GP, Terranova V and Garbisa S (1981) Effect of plasminogen activator, plasmin and thrombin on glycoprotein and collagenous components of basement membrane. *Cancer Res* **41**: 4629–4636

Ossowski L, Russo-Payne H and Wilson EL (1991) Inhibition of urokinase-type plasminogen activator by antibodies: the effect on dissemination of a human tumour in the nude mouse. *Cancer Res* **51**: 274–281

Pedersen H, Grondahl-Hansen J, Francis D, Osterlind K, Hauser HH, Dano K and Brünner N (1994) Urokinase and plasminogen activator inhibitor type I in pulmonary adenocarcinoma. *Cancer Res* **54**: 120–123

Pyke C, Kristensen P, Ralfkiaer E, Grondal-Hansen J, Eriksen J, Blasi F and Dano K (1991) Urokinase-type plasminogen activator is expressed in stromal cells and its receptor at invasive foci in human colon adenocarcinomas. *Am J Pathol* **138**: 1059–1067

Roldan AL, Cubellis MV, Masucci MT, Behrendt N, Lund LR, Dano K, Appella E and Blasi F (1990) Cloning and expression of the receptor for human urokinase plasminogen activator, a central molecule in cell surface, plasmin dependent proteolysis. *EMBO J* **9**: 467–474

Saeki T, Stromberg K, Qi CF, Gullick WJ, Tahara E, Normanno N, Ciardiello F, Kenney N, Johnson GR and Salomon DS (1992) Differential immunohistochemical detection of amphiregulin and cripto in human normal colon and colorectal tumours. *Cancer Res* **52:** 3467–3473

Schmalfeldt B, Kuhn W, Reuning U, Pache L, Dettmar P, Schmitt M, Jänicke F, Höfler H and Graeff H (1995) Primary tumour and metastasis in ovarian cancer differ in their content of urokinase-type plasmiogen activator, its receptor, and inhibitors types 1 and 2. *Cancer Res* **55**: 3958–3963

Schmitt M, Jänicke F and Graeff H (1992) Tumour associated proteases. *Fibrinolysis* **6**: 3–26

Takai S, Yamamura M, Tanaka K, Kawanishi H, Tsuji M, Nakane Y, Hioki K and Yamamoto M (1991) Plasminogen activators in human gastric cancers: correlation with DNA ploidy and immunohistochemical staining. *Int J Cancer* **48**: 29–27

Takeuchi Y, Nakao A, Harada A, Nonamy T, Fukatsu T and Takagi H (1993) Expression of plasminogen activators and their inhibitors in human pancreatic carcinoma: immunohistochemical study. *Am J Gastroenterol* **88**: 1928–1933

Vassalli J-D, Sappino AP and Belin D (1991) The plasminogen activator/plasmin system. *J Clin Invest* **88**: 1067–1072

Verde P, Stoppelli MP, Galeffi P, DiNocera P and Blasi F (1984) Identification and primary sequence of an unspliced human urokinase poly(A)+ RNA. *Proc Natl Acad Sci USA* **81**: 4727–4731

Yamanaka Y, Friess H, Büchler MW, Kobrin MS, Beger HG and Korc M (1993*a*) Coexpression of epidermal growth factor receptor and its ligands is associated with enhanced aggressiveness of human pancreatic cancer. *Anticancer Res* **13**: 565–570

Yamanaka Y, Friess H, Büchler MW, Beger HG, Uchida E, Masahiko O, Kobrin MS and Korc M (1993*b*) Overexpression of acidic and basic fibroblast growth factors in human pancreatic cancer correlates with advanced tumour stage. *Cancer Res* **53**: 5289–5296

Yamanaka Y, Friess H, Büchler MW, Kobrin MS, Kunz J, Beger HG and Korc M (1993*c*) Overexpression of HER2/neu oncogene in human pancreatic carcinoma. *Human Pathol* **24**: 1127–1134

Yokoyama M, Ebert M, Funatomi H, Friess H, Büchler MW, Johnson GR and Korc M (1995*a*) Amphiregulin is a potent mitogen in human pancreatic cancer cells: correlation with patients' survival. *Int J Oncol* **6**: 625–631

Yokoyama M, Funatomi H, Kobrin MS, Ebert M, Friess H, Büchler MW and Korc M (1995*b*) Betacellulin, a member of the epidermal growth factor family, is overexpressed in human pancreatic cancer. *Int J Oncol* **7**: 825–829

© Cancer Research Campaign 1997